# The Influence of Overeducation on Chinese Workers’ Job Satisfaction from China Household Tracking Survey (2014–2018)

**DOI:** 10.3390/ijerph192316032

**Published:** 2022-11-30

**Authors:** Wenbo Ma, Jongnam Baek, Meng Qi, Junjie Li, Bangfan Liu

**Affiliations:** 1 College of Education, Woosuk University, Wanju 55338, Republic of Korea; 2 Hebei Public Policy Evaluation and Research Center, Qinhuangdao 066004, China; 3 School of Public Administration, Yanshan University, Qinhuangdao 066004, China

**Keywords:** overeducation, job satisfaction, wage income

## Abstract

Overeducation means that the rapid growth in the numbers of secondary and higher education graduates begins to exceed the actual demand of the labor market due to this excessive expansion of education. Consequently, educated workers are faced with knowledge unemployment, or are engaged in jobs that do not match their academic qualifications, resulting in a decline in income and a waste of educational resources. In order to explore the effect of overeducation on workers’ job satisfaction, we selected data from China Household Tracking Survey (CFPS) and conducted a fixed-effect ordered logit model regression analysis. It was found that overeducation has a negative impact on employees’ job satisfaction and an impact on wage penalty. Wage income has a mediating effect on the relationship between overeducation and job satisfaction. We present three policy suggestions: for the Government’s administration department, it is necessary to actively create an environment for matching education and occupation; to improve the possibility of matching education and occupation; and to reduce the negative effect of labor contracts on the improvement of human capital and the job satisfaction of overeducators by adjusting the flexibility and stability of the labor contract. For institutions of higher learning: it is necessary to make forward-looking adjustments to the educational structure, according to the actual needs of economic and social development to adapt to the social demand for talent and development trends; to train highly skilled and high-quality workers needed for social development; and to reduce the unreasonable distribution of resources caused by overeducation. For enterprises: employees should be guided to correctly understand the unpredictable relationship between education and work and reasonably reduce the job expectations of new employees, according to their own work experience and technical level.

## 1. Introduction

“Education is the foundation of people’s livelihood, and employment is the foundation of people’s livelihood.” The development of education and employment is related to national security and stability. In the 21st century, our primary task is to improve people’s living standards, strengthen social development, and highly prioritize employment. The 19th National Congress called for building China into a major country in education, improving the quality of employment, improving people’s living standards, and promoting all-round development. Although China’s education, especially higher education, has made great progress regarding both quantity and quality in recent years, the incompatibility between education and work, especially the insufficient allocation of educational resources, has seriously affected the job satisfaction of workers, resulting in the instability of the labor market [1]. This is mainly reflected in the following aspects: generally speaking, the oversupply of education leads to a decline in the overall rate of return to education, and workers also have negative emotions at work because their talents are not brought into play. The overall job satisfaction of employees is low [2]. In some industries, there is a shortage of highly skilled personnel with many vocational skills; “higher vocational low employment” seriously hinders the realization and career development of vocational skilled personnel, posing a serious threat to their physical and mental health.

Overeducation generally means that the level of education of workers exceeds the required degree of work, and the use of labor resources becomes inefficient [3,4,5]. Accordingly, when the actual time that the labor force spends in education matches the length of experience required for work, this is called educational adaptation. Improving the quality of employment is not only the main task of national economic development, it is also the focus of a national development strategy, and more importantly, meets people’s demands for a better life [6]. Job satisfaction is a kind of comprehensive quality of work measurement method [7]. It can reflect work under the condition of excessive education to comprehensively evaluate the general effect of this education, and it can more accurately predict the behavior and performance of employees’ work and the labor market, providing an important basis and effective information for better management [8]. Therefore, when exploring the effect of overeducation, this paper takes job satisfaction as the outcome variable. In addition, wage income is not only affected by overeducation, but also plays a role in job satisfaction, so it is likely to be the action mechanism of overeducation affecting job satisfaction. Therefore, attention should be paid to its role. Based on the data of China Household Tracking Survey (CFPS) from 2014 to 2018, this paper uses the logit model of fixed effects to study the effect of overeducation on job satisfaction and its mechanism.

## 2. Literature Review

In recent years, research on the relationship between overeducation and job satisfaction in China has gradually attracted the attention of scholars. Between 2001 and 2022, 34 papers were published. In these papers, the relevant keywords or subject headings involved include: overeducated (18, refers to being used 18 times; the same as below), labor force (3), college graduates (3), education-job matching (2), employment satisfaction (2), employment quality (2), labor market (2), job matching (2), regional economic structure (2), Jiangsu Province (2), education mismatch (2), research review (2), provincial universities (2), empirical study (2), learning situation (1), comparative advantage (1), Korean students (1), matching degree (1), over-qualification (1), work engagement (1), etc. Among these literatures, 11 papers have been cited 5 times or more, namely, Does Educational Mismatch Affect Job Satisfaction—Mechanism Analysis and Empirical Test [8], and the Impact of Educational Mismatch on Employment Quality: An Empirical Analysis Based on China Household Tracking Survey Data (CFPS) [9]. The Current Situation of Education and Job Matching and Its Effect in China [2], The Study on the Labor Market Effect of China’s Over-Education [10], The Measurement of Over-Education of College Graduates and Its Impact on Employment Quality [11], The Study on the Problem of Over-Education of Chinese College Students [12], “The Influence of Education-Job Matching Degree on the Employment Satisfaction of College Graduates” [13], “The Influence of Over-education on the Employment of College Students: An Analysis from the Perspective of Comparative Advantage” [14], “Research on the Problem of Over-education in China’s Labor Market” [15], “Mobility and Job Matching” [16], and Review on the Mechanism of Overeducation on Employee Job Performance [17]. Since the beginning of this year, two papers have been published in core journals, namely, “Youth Job-Education Matching, Career Expectations and Job Satisfaction” [18] and “A Study on the Employment Quality Effect of education Matching” [19]. These papers are the main theses of our current research on the relationship between excessive education and job satisfaction. From the perspective of these thesis topics, they mainly involve two themes, namely, higher education and students’ employment matching, and the overeducation of college graduates. From this perspective, current research mainly focuses on the overeducation and employment of college students, and there remains a lack of research on the overeducation of ordinary workers. In this sense, this study has a certain theoretical value.

## 3. Research Hypotheses

Job satisfaction generally refers to an employee’s level of physical and mental satisfaction with their work environment, or their emotions and attitudes toward their work and work environment [20,21,22]. According to different research goals and objectives, scholars have many interpretations of job satisfaction, among which the most representative interpretations are the comprehensive definition and desired definition of job satisfaction. According to the comprehensive definition, job satisfaction refers to the overall evaluation of employees after weighing satisfaction and dissatisfaction with the job structure [23,24,25,26,27]. The desired definition is that job satisfaction depends on the difference between the desired value and actual value in the work environment. When the desired value is greater than the actual value, the employee’s job satisfaction improves [28,29]. In the past, there have been two main theories on the relationship between overeducation and job satisfaction based on the exceptional definition [30]. The first is incentive theory, which argues that the low job satisfaction of overeducators is caused by the failure of their job to meet their expectations [31]. In other words, under the state of overeducation, they cannot give full play to the knowledge and skills that they have learned, and thus cannot meet their job aspirations. This creates a strong sense of frustration, reduce work enthusiasm and job satisfaction [30]. The second is the theory of relative deprivation: when overeducators and educational adaptors engage in similar work, their perceived “target entity” does not match the perceived and referable resources of generalized people, which leads to dissatisfaction with workers’ rights [31]. Job demand–control theory, based on the comprehensive definition of job satisfaction, argues that employees, as “economic men”, will make trade-offs between job demands and job control. Workers who tend to have lower job demands and stronger job control will actively engage in overeducation at work. If overeducation leads to less job demands and higher job control, it will make workers more autonomous in their study and improve their efficiency and confidence in facing job and time pressure. In order to compensate for or exceed the negative effect of overeducation on workers’ job satisfaction, the effect of overeducation on job satisfaction should be weak, or even positive [32]. There are also different performances in empirical studies. Due to the differences in empirical research methods, research data and specific economic circumstances of different countries, the empirical research results of overeducation on employee job satisfaction are also quite different. The existing survey results show that both overeducation and undereducation will lead to a decline in employee job satisfaction [33]. This paper argues that educational adaptation is the best use of human capital advantage by workers to obtain more material and immaterial resources. If the educational background matches the job demand, the relationship between employees and enterprises will generate mutual attraction and choice because of the same, and then improve the job satisfaction of employees [34]. Overeducation will not only cause a waste of human resources, but also restrict the space for their development, hinder fair employment, harmonious employment relations, and reduce work enthusiasm and satisfaction [35]. Therefore, incentive theory and relative deprivation theory are more suitable for studying the effect of overeducation on job satisfaction. Therefore, this paper proposes Hypothesis 1: Compared with educational adaptation, overeducation has a negative impact on workers’ job satisfaction.

Corresponding to the first hypothesis, from the perspective of the allocation theory between education and work, work allocation is not a random process: choosing the range of office work to seek the best environment for workers; the marginal output by the double limit of individual characteristics and job characteristics; and human capital or operating characteristics, cannot fully explain the compensation effect of the mismatching of education [35]. In this model, excessive education can earn a positive reward. Workers can and will fully exert their own knowledge and technology, but due to the existence of “ceiling” work, will restrict the full play of productivity, thus resulting in a decline in the utilization of education resources. Jobs that require a higher level of education can improve labor productivity, and often have higher wages. Compared with other theories, allocation theory provides a better explanation for the educational mismatch in China. Therefore, this paper puts forward Hypothesis 2: Overeducation has a wage penalty effect.

There are also many connections between salary and job satisfaction. High efficiency salary can reduce the dimission rate, improve job satisfaction, and improve work performance. Wage income is a kind of value signal given to employees by the company. It is not only an affirmation of their work ability and performance, but also their status in the distribution of compensation. Therefore, increasing wages can effectively improve employees’ pride and meet their basic psychological needs for work. Therefore, this paper puts forward Hypothesis 3: Wage income has a significant positive impact on job satisfaction.

In conclusion, overeducation has a direct impact on employees’ job satisfaction and an indirect impact through wage income. It can be seen that wage income plays a certain mediating role in the effect of overeducation on employees’ job satisfaction. Therefore, this paper puts forward Hypothesis 4: Wage income plays a mediating role between overeducation and job satisfaction.

**Figure 1:**
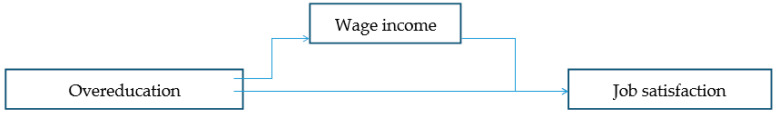
Theoretical architecture.

Figure 1 shows the theoretical framework of this paper. Based on previous analysis, this article explores the impact of overeducation on employee job satisfaction and the mediating effect of wage income to better reveal its internal mechanism.

## 4. Model and Data

### 4.1. Model Building

In order to investigate the direct effect of overeducation on employees’ job satisfaction, this study established the following econometric model according to Equation (1):

Wsf_it_ = α_0_ + α_1_Oedu_it_ + α_2_Uedu_it_ + α_3_X_it_ + μ_i_ + v_t_ + ε_it_ (1)

where the explained variable Wsf is the employee’s job satisfaction, the main explanatory variable Oedu is the overeducation dummy variable, X is the control variable, I and t are individuals and years, α is the parameter to be estimated, μ and ν are individual and time fixed effects, and εit denotes the random disturbance term.

As can be seen from the previous analysis, excessive education will indirectly affect employees’ job satisfaction through wage income. Therefore, this paper adopts the stepwise regression method of mediating effect model and the mediation effect test step of a discrete ordered dependent variable to verify this influencing mechanism. In the first step, Model (1) is utilized, where the explained variable is job satisfaction, the explanatory variable is overeducation, and the overall effect of overeducation on job satisfaction is investigated. In the second step, the explained variable is log wage income, the explanatory variable is overeducation, and the effect of overeducation on employee wage income is investigated. In the third step, on the basis of Model (1), log wage income is added as the intermediary variable to investigate the influence of overeducation and wage income on job satisfaction. For these variables, the regression model of the second and third steps can be expressed as:

Lnwage_it_ = β_0_ + β_1_Oeduit + β_2_Uedu_it_ + β_3_X_it_ + μ_i_ + v_t_ + ε_it_ (2)

Wsf_it_ = γ_0_ + γ_1_Oedu_it_ + γ_2_Uedu_it_ + γ_3_Lnwage_it_ + γ_3_X_it_ + μ_i_ + v_t_ + ε_it_ (3)

In Equations (2) and (3), Lnwage is log wage income, β and γ are parameters to be estimated. If α_1_ in Equation (1) is significant, and β_1_ and γ_3_ are also significant, then overeducation affects employees’ job satisfaction by affecting wage income. Moreover, if β_1_, γ_3_ and γ_1_ are the same, then wage income mediates the relationship between overeducation and job satisfaction. Moreover, if γ_1_ is significant, then wage income is part of the mediating variable; If γ_1_ is not significant, then wage income is the full mediator variable. If at least one of β_1_ and γ_2_ is not significant, the Sobel test is required for further determination.

The explained variable job satisfaction in this paper is an ordered discrete variable with a small number of categories and effectively eliminates the endogeneity bias caused by individual heterogeneity that does not change over time. Therefore, for the estimation of Equations (1) and (3), the fixed effects ordered logit model is used to estimate the parameters.

### 4.2. The Data Source

Data from the 2014 and 2018 adult databases of the China Household Tracking Survey (CFPS) (http://www.isss.pku.edu.cn/cfps/index.htm, (accessed on 22 October 2022)) were used in this study. On the basis of the research purpose, the following points were considered during the recruitment of participants: (1) participants whose employment status in 2014 and 2018 was on-the-job and whose type of employment was non-agricultural employment; (2) participants aged 16–60 years in the labor force with weekly working hours of between 20 and 112 h; (3) participants whose annual salary is less than CNY 5000 and whose monthly income is in the highest and lowest 1% were excluded; (4) participants with illiterate education level were excluded; and (5) participants with invalid or missing data, for whom logic errors and outliers were removed. In this paper, the cross-sectional survey data collected between 2014 and 2018 were merged into balanced panel data.

The data variables in this article are derived from the original data of CFPS. How the original data was obtained is not published, so we do not know. However, in general, the original data is obtained through questionnaires. In the questionnaire, for example, there were such questions as: “From the perspective of knowledge and skills, how high do you think the actual education of this job is needed?” The relevant data information of related issues is indirectly judged on whether the investigator has undergone excessive education. The main problem discussed in this article lies in the impact of excessive education on work satisfaction and its causes, and the investigation of education and work adaptation of that education has not yet been studied.

### 4.3. Variable Selection

#### 4.3.1. Explained Variable

Job satisfaction (Wsf): In line with research goals, this paper combines the “very dissatisfied” and “not very satisfied” in the original CFPS classification with “dissatisfied”, which is represented by 1, and re-represents the “general”, “relatively satisfied” and “very satisfied” in the original classification by 2, 3 and 4. After reassignment, the skewness of job satisfaction is close to 0, and kurtosis is close to 3, which essentially meets the requirements of normal distribution.

#### 4.3.2. Explanatory Variable

Overeducation (Oedu): In this paper, the situation that the education level required by the work is equal to the actual education level of workers is called educational adaptation, and the situation that the education level required by the actual work is lower than the actual education level of the workers is called overeducation. Overeducation is denoted by 1; others are denoted by 0.

#### 4.3.3. Mediating Variable

Log wage income (Lnwage): This study represents the income level of employees as an hourly wage, and according to the convention, takes the logarithm. Additionally, the measured hourly wage = monthly wage income/(average number of hours worked per week × number of weeks worked per month).

### 4.4. Descriptive Statistics

Table 1 shows the descriptive statistical results of the main variables, and Table 2 shows the statistical results of job satisfaction of educational adaptors and overeducators. The results shown in Table 2 indicate that: (1) the overall job satisfaction of employees is at a medium level, with a relatively high job satisfaction of “relatively satisfied” and “general” and relatively low job satisfaction of “dissatisfied” and “very satisfied”; (2) the overall job satisfaction of education adaptors and overeducators in 2018 was higher than that in 2014, and the job satisfaction increased from 2014 to 2018; (3) the job satisfaction of overeducators was lower than that of education adaptors, and the difference was significant between 2014 and 2018. The above conclusion shows that the job satisfaction of overeducators is lower than that of education adaptors, and overeducation may make their job satisfaction decrease, which is consistent with the expectation assumed in this study.

## 5. Empirical Results and Regression Analysis

### 5.1. Test of the Comprehensive Effect of Overeducation on Job Satisfaction

This paper first investigates the comprehensive effect of overeducation on job satisfaction of employees. For the orderly logit Model (1) and the use of the fixed-effects regression, the results are shown in Table 3 in the first column (1). Excessive education at a 5% statistical level has clear negative effects on employees’ job satisfaction and level of education as opposed to some other groups, for whom excessive education would contribute to their job satisfaction. The conclusion of Hypothesis 1 is verified by the fixed-effects ordered logit model, which indicates that overeducation has a significant negative effect on the job satisfaction of employees compared with education adaptors with the same education level.

### 5.2. The Mediating Effect Test of Wage

In this paper, logarithmic wage is used as the mediating variable to conduct a regression analysis in Equations (2) and (3). In the data of the fixed effects model in column (2) of Table 3, the typical wage penalty effect of overeducation is calculated. Under the same level of education, the wage income of overeducated people is lower than that of the educated people, which verifies the observation of Hypothesis 2. The regression results in column (3) of Table 3 show that in the fixed-effects ordered logit model, overeducation and wage income are both significantly negative and positive, indicating that wage income has a mediating effect on the influence of overeducation and job satisfaction, and it is partially mediating. This indicates that, compared to people with a similar education level, overeducation will not only have a negative impact on employees’ job satisfaction, but also indirectly affect employees’ job satisfaction through wage income, and the mechanism of “overeducation-wage income—job satisfaction” has been significantly established.

In order to further test the robustness of the mediating effect of wages, the regression coefficients obtained were transformed into a unified scale using standardized methods, and then the correlation between variables was analyzed using the Sobel test. It is found that the Sobel test rejects the null hypothesis of no mediating effect at a significance level of 1%, proving that part of the effect of overeducation on job satisfaction comes from wages. Therefore, Hypotheses 3 and 4 are verified.

### 5.3. Significance Analysis

Ols is ordinary least squares and is the most fundamental form of regression analysis. Ordered logit belongs to a sort selection model in which error distribution follows logical distribution. Through the F test, the values of the F/Chi2 of (1), (2), (3) in the fixed effects in Table 3 by recalculating of the fixed effects in Table 3 are 443.67 ***, 15.55 ***, 396.19 ***, respectively (note: ***, represent 1%, significant level). All three values are high, indicating high significance, which indicates that Hypotheses 1, 2, and 3 are valid.

In addition to treating work satisfaction as the order number value, an orderly logit model is used to estimate the effect of education mismatch on the work satisfaction of the workers, and there is also a measurement method to regard the work satisfaction as the base value. regression analysis. Based on this, the satisfaction of the worker is also regarded as the base value, and the fixed effect model is used for inspection. The results show that whether the working satisfaction of the worker is regarded as the base value or the order value, the main conclusions obtained by the logit model in this article are basically the same.

In order to test the accuracy and reliability of the results, this article adds some variables that reflect the work protection of workers in the control variables, and mainly include: whether they enjoy employee pension insurance; whether they enjoy employee medical insurance; and whether they enjoy a housing provident fund. After joining other control variables, the core of the core explanation variables and significant changes in this article are small. The impact of the newly added control variable on the working satisfaction of the workers is not significant, indicating that the model setting of this article is reasonable to a certain extent.

## 6. Conclusions

Data from a study on Chinese families (CFPS), collected between 2014 and 2018, were used to clarify the mismatching of education and the relationship between job satisfaction and mechanisms of action from the perspective of excessive education. Using a fixed-effects ordered logit model, and the mediation effect testing method, the mechanism of action of the influence of excessive education job satisfaction was further discussed. The findings are as follows: (1) under the same education level, overeducation reduces the job satisfaction of workers; and (2) overeducation has a wage penalty effect, and wage income plays a mediating role between overeducation and job satisfaction. However, overeducation is a long-term and dynamic process. This paper investigates the effect of overeducation on job satisfaction based on CFPS data from 2014 and 2018, which have certain limitations.

According to the above analysis, from the perspective of the allocation theory of education and work, the allocation efficiency of China’s labor market is low, the problem of excessive education is prominent, and the mismatch of jobs is serious. Additionally, the job is the dominant factor determining the income and job satisfaction of laborers in China’s labor market, which seriously leads to the severity of incentive failure and relative deprivation. This leads to a structural dislocation in the workforce. Studies have shown that mobility can reduce the possibility of over-education of workers and increase their chances of finding jobs that are more compatible with their education and with higher satisfaction. Compared with non-mobility and other types of mobility, multiple mobility has the greatest effect on job matching [9]. However, due to various economic, social, institutional, and other reasons, the work flow of our workers has not formed a benign structure, especially for those workers working in the national civil service system, public institutions, and state-owned enterprises, it is difficult to achieve mobility, let alone multiple mobility.

Through previous analysis, we have verified the hypothesis that “excessive education has wage penalty effect”. The fact shows that the overeducated bear the wage penalty effect, while the undereducated get the wage premium [9]. Such a situation not only has a negative impact on educational incentives, but also directly weakens job satisfaction.

Through the previous analysis, we also verified the hypothesis that “compared with education adaptation, excessive education has a negative impact on workers’ job satisfaction”. In fact, relevant domestic studies also show the following three points: first, overeducated people have lower job satisfaction, while undereducated people have higher job satisfaction. Second, from the perspective of education level and urban and rural areas, the impact of educational mismatch on the quality of employment is more significant in the highly educated group and urban residents. In the group with a low education background, the impact of education mismatch on income is small; the higher the education background, the greater the impact of education mismatch. Compared with rural residents, the education mismatch among urban residents has a higher impact on job satisfaction, that is, the wage income of urban residents is greatly affected by the education mismatch. It indicates that while cities provide more jobs, more over-educated people are engaged in jobs inconsistent with their educational background, which reflects the higher degree of job competition in cities and the lower rate of return on education [2]. Third, due to the relative relationship between job mobility and educational attainment, there are no significant regional differences. Compared with the east, workers in the central and western regions have less job mobility and higher education. Compared with the central and western regions, the mobility of workers in the eastern region is greater, and the workers have lower education. In this way, the differences between mobility and education are complementary, and in terms of the matching degree of workers’ jobs, there is no great difference between our midwest.

## 7. Suggested Countermeasures

Since the reform and opening up in China, the large-scale expansion of the education system has rapidly increased the average education level of workers. However, the mismatch between education and employment has become increasingly serious, and is the main factor affecting workers’ income and reducing job satisfaction. 

Therefore, based on the previous analysis and from the perspective of the incentive theory, relative deprivation theory, and allocation theory, the following three countermeasures are proposed:

First of all, for the country, it is necessary to introduce timely and effective policy measures to enhance the matching degree between education and the work of workers. When China’s education system, especially at levels of higher education, reaches a certain size, education quality notably improves and encourages fair competition to reduce the risk of education as an unreasonable allocation. This carries out the role of “maximize talent”, improving the allocation of human resources, and employee job satisfaction plays a major role. The Government should: actively create favorable conditions for education and career matching, including establishing and perfecting the talent exchange platform to reduce barriers; encourage enterprises to open recruitment information and hiring processes; emphasize the industry structure, education structure, and coordinated development employment structure; cultivate human capital; rationally allocate education resources; and improve education and the possibility of a professional match. The Chinese Government should also improve China’s labor contract law by adjusting the flexibility and stability of labor contracts to reduce their negative effect on the improvement of human capital and the job satisfaction of overeducators.

Secondly, educational institutions should continue to improve the education and teaching mechanism, focusing on improving the knowledge and ability of workers to adapt to the labor market. Higher education institutions should be subject to innovative reforms according to the actual needs of China’s economic and social development. This requires a highly skilled but reduced workforce because of the unreasonable allocation of higher education resources. At the same time, in order to reduce the negative rate of formal education, the majority of staff and workers, especially those with higher education backgrounds, should be sure to update knowledge structures after graduation, and constantly master practical skills to meet market needs.

Finally, for workers, we should strengthen publicity and guidance, strengthen vocational training, and focus on improving the quality of workers to adapt to the labor market. Young workers should be guided to correctly understand the uncertain relationship between education and work, and according to their own work experience and technical level, reasonably reduce the job expectations of new employees. At the same time, enterprises should be encouraged to reduce the probability of the educational mismatch of young employees through pre-employment tests, on-the-job training, job rotation, and other methods, so as to create a better employment development space for young employees. 

## Figures and Tables

**Table 1:** Descriptive statistics of the main variables.

Variable	Overall		In 2014		In 2018	
	Mean	Standard Deviation	Mean	Standard Deviation	Mean	Standard Deviation
Wsf	2.5546	0.7389	2.4666	0.7021	2.6426	0.7642
Oedu	0.3439	0.4751	0.3420	0.4746	0.3457	0.4758
Uedu	0.1719	0.3774	0.1706	0.3763	0.1733	0.3787
Lnwage	8.0021	0.4532	7.8712	0.4264	8.1330	0.4415

**Table 2:** Descriptive statistics of job satisfaction of education adaptors and education mismatched.

Variable Name	Percentage (%)				Mean		
	Not Satisfied	General	Relatively Satisfied	Very Satisfied	Overall	In 2014	In 2018
Education matches	6.62	35.88	49.02	8.48	2.5937	2.4870	2.7017
Excessive educators	9.19	42.12	42.65	6.04	2.4554	2.3852	2.5248
Overall	7.08	38.23	46.84	7.85	2.5546	2.4666	2.6426

**Table 3:** The effect of overeducation on job satisfaction and the mediating effect test.

Variable		Fixed Effects		
		Ordered Logit	OLS	Ordered Logit
		Job Satisfaction	Wage	Job Satisfaction
		Formula (1)	Formula (2)	Formula (3)
Oedu	beta	−0.6587 **	−0.0982 **	−0.6299 **
	standard error	0.3009	0.0395	0.2981
Uedu	beta	−0.8050 *	0.1025 *	−0.8728 **
	standard error	0.4622	0.0549	0.4420
Lnwage	beta			0.8341 ***
	standard error			0.2419

Note: ***, ** and * indicate significance levels of 1%, 5% and 10%, respectively, with robust standard errors in parentheses. Beta is the regression coefficient.

## Data Availability

The data used to support the findings of this study are available from the corresponding author upon request.
